# Role of antenatal care and iron supplementation during pregnancy in preventing low birth weight in Nepal: comparison of national surveys 2006 and 2011

**DOI:** 10.1186/2049-3258-72-4

**Published:** 2014-02-05

**Authors:** Vishnu Khanal, Yun Zhao, Kay Sauer

**Affiliations:** 1 Maternal and Child Health Consultant, Sauraha Pharsatikar-1, Rupandehi, Nepal; 2 School of Public Health, Curtin University, Perth, Australia

**Keywords:** Antenatal care, Iron supplementation, Low birth weight, Nepal

## Abstract

**Background:**

Low birth weight (LBW) is a major cause of neonatal deaths in developing countries including Nepal. Its social determinants in Nepal have rarely been identified. This study aimed to identify the factors associated with low birth weight among under-five children comparing data from the Nepal Demographic and Health Surveys (NDHS) of 2006 and 2011.

**Methods:**

Pooled data from the Nepal Demographic and Health Surveys (NDHS) of 2006 and 2011 were analysed initially and the two survey data were then compared separately. The association between LBW and socio-demographic and health related factors were analysed using multiple logistic regression analysis with a stepwise backward elimination procedure. Complex Sample Analysis method was used to account for study design and sampling.

**Results:**

A total of 2845 children, 923 children in 2006 and 1922 children in 2011, had their birth weight recorded. The mean birth weight was 3024 (SD = 654.5) grams. A total of 12.1% (95% Confidence interval (CI); 10.6%-13.7%) children had low birth weight (<2500 grams) at the time of birth. Attending antenatal care was found to be consistently associated with low birth weight for the pooled survey data, and both 2006 and 2011 survey data, respectively. Not attending antenatal care increased the odds of having a LBW infant by more than two times [OR 2.301; 95% CI (1.526-3.471)]. Iron supplementation, which is an integral part of antenatal care in Nepal, was also significantly associated with birth weight for combined and individual surveys. Mothers not consuming iron supplementation during their pregnancy were more likely to have LBW infants [OR 1.839; 95% CI (1.282-2.363)]. Residing in the Far-western and Eastern region were also significant risk factors for LBW in the pooled dataset and in 2011 survey.

**Conclusions:**

The current study indicated there was no significant decrease in the LBW prevalence and there is a need of targeted interventions aimed at decreasing the high rate of LBW through increasing antenatal care and consumption of iron supplementation during pregnancy.

## Background

Low birth weight (LBW) is one of the risk factors for neonatal mortality which increases the odds of deaths by 20-30 times [1]. A birth weight less than 2500 gram is defined as low birth weight irrespective of the weeks of gestation [2]. The low birth weight infants are at risk of developing cerebral palsy, or more susceptible to infection in short run and they are more likely to develop breathlessness, physiological immaturity and lower weight and shorter stature in long term [2,3]. Poor social adaptation in school and other settings has also been reported among the LBW infants when they are grown [3-6].

The prevalence of LBW is around 15% in developing countries [3]. However, in many developing countries, the majority of births occur in home, therefore, the information on birth weight is not available. For those countries the Demographic and Health Surveys (DHS), conducted every five years, are the sources of population health indicators. In these surveys, birth weight is recorded based on mother’s recall or the birth certificate and the prevalence of LBW is reported as an important indicator of neonatal health [7,8].

Risk factors for LBW have been of interest for researchers over a long period. As many as 50 risk and protective factors have been identified by different reviews on LBW infants. Genetic make up, demographic factors, maternal nutritional factors, obstetric factors, maternal health condition and service utilisation are some of the factors that have been of recent interest [7,9]. Maternal health status and the use of antenatal care (ANC) service during pregnancy have been reported to be one of the major determinants of birth weight [9,10]. ANC provides an opportunity for a pregnant woman to have her health checked, manage any problems that arise during pregnancy and obtain counselling services. Counselling advice to pregnant woman revolves around taking adequate rest, reducing physical workload, and eating adequate nutrition including iron-folic acid supplementation in Nepal [11]. In Nepal, iron-folic acid supplementation is provided at no cost at government health facilities throughout the country [11]. An earlier double blinded cluster randomised study from the Eastern Nepal reported the beneficial effect of iron-folic acid supplementation during pregnancy in reducing LBW [relative risk: 0.84; 95% CI (0.072-0.99)] showing an increase in the mean birth weight by 37 grams [12].

Nepal is one of the exemplary countries successful in reducing the child and maternal mortality in this century. However, recent Nepal Demographic and Health Survey (NDHS) 2011 showed that neonatal death rate remained stagnant (33 per 1000 births) since 2006 despite having tremendous efforts from the Government of Nepal to reduce neonatal, infant and child deaths [8,13]. In part, a higher prevalence of low birth weight of 12% (nationally) to as high as 28% in some parts of country could be one of the many reasons of such higher neonatal deaths [8]. An extensive search on the major databases did not yield any previous report on the factors associated with LBW in Nepal based on the community based survey covering the entire country. An updated knowledge on the factors contributing the higher prevalence of LBW will enable to design a better public health intervention and contribute in child survival in Nepal. This study aimed to (i) identify the factors associated with low birth weight and (ii) compare factors associated with low birth weight among under-five children between 2006 and 2011.

## Methods

The Nepal Demographic and Health Surveys (NDHS) of 2006 and 2011 [8,13] were nationally representative cross sectional studies based on multistage cluster sampling conducted every five years. In first stage, the primary sampling units (wards in rural and sub wards in urban areas) were selected. In second stage, households were selected by a random selection of households from the wards. Details of clustering, listing, and sample selection have been explained elsewhere (11, 12). The intended sample in the 2006 survey (12) was 8600 women aged 15-49 years. A total of 4397 men in 2006, aged 15-59 years were interviewed from every second selected household. The 2011 survey interviewed 12,674 women and 4,121 men (11). The response rate was 96% in 2006 and 95.3% in 2011. Three sets of internationally validated questionnaires were used to collect different levels of information: (i) household information–covered information about all the members of the household; (ii) women’s information; and (iii) men’s information (11, 12).

This study utilised the 2006 and 2011child datasets that contained information on under-five children. The datasets contained information on the child, mother, father, and household characteristics necessary for further analysis. Only those cases with recorded birth weight were included in the analysis. Multiple births were excluded from the study as this have been reported to be a known risk factors of LBW and may mask the effect of other socio-demographic variables due to its stronger effect on birth weight [14,15]. The children without a recorded birth weight were also excluded from the analysis.

### Conceptual framework

**Figure 1:**
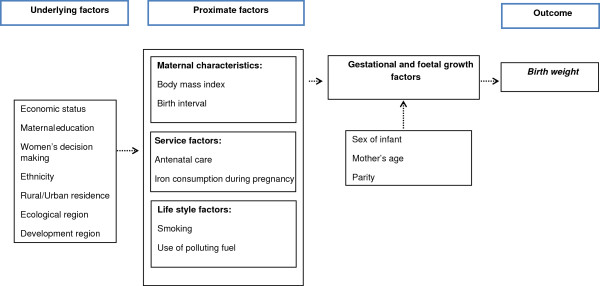
**Potential determinants of low birth weight. **Source: Adapted from: Dharmalingam et al. 2009 [16]. All variables included in analysis.

To conceptualise the analysis, we adapted the framework used by Dharmalingam et al. [16]. Figure 1 illustrates the potential causal link to LBW in Nepal. In this framework, Dharmalingam et al. [16] suggested that LBW is directly or indirectly caused by three major factors: Underlying factors (maternal socio-demographic characteristics), Proximate factors (maternal characteristics such as body mass index, service use, birth interval, smoking, use of type of cooking fuel), and Gestational and foetal growth factors (sex of infants, mothers age and parity). In our study, the selection of factors were based on previously published studies including Dharmalingam et al. [16] and others [7,17].

### Definition of variables

Birth weight <2500 grams is defined as LBW. Birth weights recalled by mothers or recorded from the birth card were included for analysis in this study. Birth weight was categorised into LBW (<2500grams) and normal birth weight (>=2500grams), which serves as the dependent variable in our study.

Independent variables were selected based on the framework adapted for analysis. Categories of the independent variables were based on previously published NDHS data based studies [18] and other developing countries using similar DHS datasets [7,16]. Five development regions described as Eastern, Central, Western, Mid-western and Far-Western region for administrative purpose; Ethnicity was classified as (i) relatively advantaged–Brahmin, Chhetri, Thakuri, Gurung, Newar, and Sanyasi, (ii) relatively disadvantaged–Janjati including indigenous groups and (iii) relatively disadvantaged-Dalit [19]. Among all caste groups, Dalits have traditionally experienced high level of social exclusion and marginalisation in Nepal. Cooking fuel was categorized as (i) relatively non-polluting: biogas, electricity, natural gases, LPG and (ii) relatively polluting: kerosene, coal, ignite, charcoal, wood, straw, agricultural crop, animal dung [7]; ANC visits were initially recorded as continuous variable which is then re-categorised into (i) no ANC visit (ii) one to three ANC visits, and (iii) four or more ANC visits; Birth order was categorized into three categories; (i ) first (ii) second or third, and (iii) fourth or higher; Birth interval/spacing of the index child to previous child was categorised as (i) no previous birth, (ii) less than 24 months apart and (iii) 24 months or more; Body mass index (BMI) was calculated by using height (in meter) and weight of mothers (in kilogram) measured at the time of survey. BMI was categorised into three categories based on Asian standard (i) underweight (<18.5 kg/m^2^) (ii) normal (18.5-23.00 kg/m^2^) and (iii) overweight (>23.00 kg/m^2^) [20]. Iron-consumption was regrouped as binary variable: as (i) consumed and (ii) not consumed. In Nepal, iron supplementation is recommended during pregnancy and provided free of cost from the public health facilities. Although NDHS asked as iron consumption, the dosage form that is supplied from public health facilities has iron-folic acid. Economic status was classified based on the wealth index [8,13]. The wealth index was categorised into five categories; (i) poorest (ii) poor (iii) middle (iv) richer and (v) richest. Details of creating the wealth index is published elsewhere [8]. Women’s ability to make a decision on health care were included as indicator of maternal autonomy and categorised into; (i) women (ii) women and husband together and (iii) husband and others.

### Statistical analysis

Two survey dataset, namely, NDHS 2006 and NDHS 2011 [21], were pooled for this study. The prevalence of LBW was reported as a percent of all birth weight recorded in the pooled dataset. Association between LBW and categorical independent variables of interest were determined first by using Chi-square test (χ^2^). All the factors included in the conceptual framework (Figure 1) which we considered important based on previous studies [7,16] were included in the multiple logistic regression to control the confounding effect of each other. Backward elimination process was used to obtain the final regression model; namely, Model 1 (pooled data), Model 2 (2006 survey data) and Model 3 (2011 survey data). Complex Sample Analysis method was used to report the prevalence of LBW and perform the regression analysis to account for the study design and sampling method [22].

Interactions between the survey period (depicted by an indicator variable: 0=2006 survey and 1=2011 survey data) and significant determinants found in Model 1 (pooled data) were then assessed. The interaction term was found significant; indicating the effects of those significant determinants on LBW are significantly different for the two surveys. Next we tested wether the associations from Model 1 were different by survey period (i.e. NDHS 2006 and 2011) thus additional models were run for NDHS 2006 (Model 2) and NDHS 2011 (Model 3). We tested the multicollinearity issue among the independent variables. ANC was highly correlated with iron supplementation during pregnancy (r = 0.88). Therefore, while building models, ANC was used along with other independent variables. In the next step, iron supplementation was introduced into the model replacing ANC from each models to check if iron supplementation was still statistically significant. Given the large number of variables, the adjusted odds ratios and their 95% confidence intervals (CI) were only reported for statistically significant variables in the final model. Additionally, independent sample t-test was used to analyse the difference between mean birth weight in 2006 and 2011. A Chi-square test (χ^2^) was used to examine the difference in the prevalence of LBW in 2006 and 2011 surveys. A p-value <0.05 was considered statistically significant. All statistical analyses were conducted using IBM SPSS Statistics for Windows, Version 19.0 (IBM Corp. Released 2010. Armonk, NY: IBM Corp USA). Ethical approval from Curtin University Human Research Ethics Committee [protocol approval–SPH-16-2012] was also obtained for the data analysis.

## Results

### Low birth weight prevalence

A total of 2845 children had their birth weight reported by mothers: 923 in 2006 and 1922 in 2011. The mean birth weight was 3024 (SD: 654.5) grams. In 2006, it was 3012 (SD = 667) grams and in 2011, it was 3030 (SD = 649) grams. There was no significant difference in the mean birth weight between 2006 and 2011[mean difference: 18grams, t-test p-value = 0.489]. The overall prevalence of LBW was 12.1% (95% CI: 10.6%-13.7%) while 13.2% (95% CI: 10.6%-16.3%) in 2006 survey and 11.5% (95% CI: 9.7%-13.6%) in 2011 survey. No statistically significant difference was found in the prevalence of LBW during the two surveys (Chi-square test p value: 0.331).

### Characteristics of participants

Table 1 presents the characteristics of 2845 mother-children pairs included in this study.

**Table 1:** Characteristics and rates of low birth weight among under five children, Nepal Demographic and Health Surveys, 2006 and 2011(N = 2845)

**Factors**	**Total [%]**	**LBW n [%]#**	**Unadjusted odds ratio**
**Survey years**		P = 0.33	P = 0.33
2006	923 (32.4)	108 (13.2)	1.00
2011	1922 (67.6)	235 (11.5)	0.855 (0.623-1.173)
**Gestational factors**			
**Sex of infant**		P = 0.387	P = 0.387
Male	1484 (52.2)	171 (11.4)	1.00
Female	1361 (47.8)	172 (12.8)	1.143 ( 0.844-1.548)
**Mother’s age**		P = 0.157	P = 0.144
15-19 years	259 (9.1)	47 (16.7)	1.552 (1.026-2.348)
20-32 years	2346 (82.5)	269 (11.5)	1.00
32 and higher	240 (8.4)	27 (12.6)	1.119 (0.641-1.955)
**Parity**		P = 0.166	P = 0.160
First	1448 (50.9)	190 (13.3)	1.00
Second or third	1152 (40.5)	124 (10.6)	0.771 (0.588-1.009)
Fourth or more	245 (8.6)	29 (11.1)	0.813 (0.486-1.362)
**Proximate factors**			
**Body mass index**		P = 0.017	P = 0.025
< 18.5 (Underweight)	291(16.0)	42 (13.6)	0.931 (0.595-1.457)
18.5-23.0 (Normal)	980 (53.7)	132 (14.5)	1.00
> 23.0 (Overweight)	553 (30.3)	46 (8.3)	0.533 (0.339-0.837)
**Birth interval**		P = 0.222	P = 0.229
No previous birth	1526 (53.6)	195 (13.0)	1.00
< 24 months	516 (18.1)	64 (12.0)	0.921 (0.641-1.298)
> = 24 months	803 (28.2)	84 (10.2)	0.757 (0.550-1.040)
**Antenatal care**		P < 0.001	P < 0.001
No ANC visit	514 (18.1)	85 (18.3)	2.079 (1.511-2.859)
1-3 ANC visit	541 (19.0)	74 (13.5)	1.443 (1.015-2.053)
4 or more ANC visit	1790 (62.9)	184 (9.7)	1.00
**Iron consumption during pregnancy**		P < 0.001	P = 0.001
No/Do not know	635 (22.3)	100 (16.8)	1.678 (1.255-2.244)
Yes	2210 (77.7)	243 (10.7)	1.00
**Smoking**		P = 0.746	P = 0.736
No	2776 (97.6)	335 (12.0)	1.00
Yes	69 (2.4)	8 (13.7)	1.165 (0.477-2.850)
**Use of fuel**		P = 0.029	P = 0.030
Relatively non polluting	866 (30.4)	79 (9.3)	1.00
Relatively highly polluting	1979 (69.6)	264 (13.2)	1.494 (1.040-2.146)
**Underlying factors**			
**Wealth index**		P = 0.576	P = 0.568
Poorest	281 (9.9)	41 (15.9)	1.00
Poor	359 (12.6)	43 (12.3)	0.759 (0.447-1.289)
Middle	453 (15.9)	67 (12.4)	0.747 (0.455-1.226)
Richer	686 (24.1)	78 (11.2)	0.663 (0.400-1.099)
Richest	1066 (37.5)	114 (11.5)	0.684 (0.430-1.090)
**Maternal education**		P = 0.018	P = 0.021
No education	664 (23.3)	83 (10.6)	1.151 (0.627-2.112)
Primary	512 (18.0)	85 (17.2)	2.011 (1.094-3.695)
Secondary	1291 (45.4)	141 (11.5)	1.251 (0.736-2.126)
Higher	378 (13.3)	34 (9.4)	1.00
**Women’s decision making for health**		P = 0.022	P = 0.030
Women	649 (22.8)	55 (8.8)	1.00
Women and husband together	992 (34.9)	110 (11.8)	1.386 (0.927-2.071)
Husband or others	1204 (42.3)	178 (14.1)	1.701 (1.146-2.526)
**Ethnicity**		P = 0.541	P = 0.550
Relatively advantaged	1620 (57.0)	190 (12.0)	1.00
Relatively disadvantaged(Janjati)	813 (28.6)	93 (11.4)	0.949 (0.690-1.304)
Relatively disadvantaged (Dalit)	411 (14.5)	60 (14.0)	1.202 (0.830-1.743)
**Place of residence**		P = 0.943	P = 0.943
Urban	1175 (41.3)	136 (12.0)	1.00
Rural	1670 (58.7)	207 (12.1)	1.011 (0.751-1.360)
**Ecological region**		P = 0.995	P = 0.997
Mountain	261 (9.2)	28 (12.2)	1.00
Hill	1112 (39.1)	138 (12.0)	0.982 (0.595-1.619)
Terai	1472 (51.7)	177 (12.1)	0.983 (0.603-1.605)
**Development region**		P = 0.022	P = 0.028
Eastern	647 (22.7)	90 (15.3)	1.700 (1.142-2.530)
Central	753 (26.5)	80 (9.6)	1.00
Western	554 (19.5)	60 (10.8)	1.137 (0.722-1.791)
Mid-western	470 (16.5)	51 (11.4)	1.208 ( 0.733-1.993)
Far-western	421 (14.8)	62 (16.7)	1.892 (1.179-3.035)

# the percent indicates for the row total of independent variables, the percentage shows the weighted percent therefore it may not be equal to raw percent, the p value indicates the probability value of chi square test.

The majority of children (82.5%) were born to mothers between the ages of 20-29 years. Six in every ten (62.9%) of the mothers attended four or more antenatal care (ANC) visits during their pregnancy. The majority (77.7%) had consumed iron supplementation at some time point of their pregnancy. Only small number (2.4%) of mothers reported smoking at the time of survey. About a quarter (22.8%) of mothers did not have formal education. The majority (58.7%) were from rural areas (58.7%). The majority of children were from Central (26.5%), Western (19.5%) and Eastern (22.7%) regions; only 16.5% and 14.8% were from Mid-western and Far-western regions, respectively.

### Factors associated with low birth weight

All the factors included in Figure 1 were reported to be important determinants of LBW in various studies. The result of the Chi-square test (χ^2^) and binary regression analyses are presented in Table 1 for all factors under study. All factors shown in Figure 1 were included in the multiple regression analysis in the pooled data (Model 1; Table 2). ANC visits, maternal education, and development region remained statistically significant after controlling other variables in conceptual framework including the indicator variable of the survey years. Mothers who had no ANC visit were twice more likely [OR 2.301; 95% CI (1.526-3.471)] to have LBW infants compared to mothers who had four or more ANC visits. When ANC was replaced by iron supplementation, iron supplementation remained statistically significant and other significant variables remained unchanged. Mothers who did not consume iron supplements during pregnancy were more likely to have LBW infants [OR 1.839; 95% CI (1.282-2.636)]. The mothers with primary education were more likely to have LBW infants [OR 1.491; 95% CI (1.024-2.171)] than their counter parts who had secondary education. The odds of having LBW infants were higher for mothers residing in the Eastern [OR 1.982; 95% CI (1.261-3.115)] and Far-western region [OR 1.910; 95% CI (1.035-3.528)] compared to mothers from Central region.

**Table 2:** Factors associated with low birth weight in Nepal: Adjusted odds ratio

**Factors**	**Model 1**	**Model 2 (NDHS 2006)**	**Model 3 (NDHS 2011)**
**Antenatal care**			
4 or more ANC visit	1.00	1.00	1.00
No ANC visit	2.301 (1.526-3.471)*	3.118 (1.628-5.696)*	1.578 (1.062-2.345)*
1-3 ANC visit	1.513 (0.948-2.412)	2.031 (1.050-3.929)	1.080 (0.700-1.665)
**Iron consumption during pregnancy**			
Yes	1.00	1.00	1.00
No/Do not know	1.839 (1.282-2.636)*	1.840 (1.087-3.116)*	1.490 (1.013-2.193)*
**Maternal education**			
Secondary	1.00	1.00	1.00
No education	0.802 (0.588-1.154)	1.027 (0.615-1.714)	0.717 (0.449-1.147)
Primary	1.491(1.024-2.171) *	2.113 (1.224-3.650)*	1.229 (0.751-2.013)
Higher	0.910 (0.525-1.578)	0.358 (0.127-1.008)	1.130 (0.611-2.092)
**Development region**			
Central	1.00	1.00	1.00
Eastern	1.982 (1.261-3.115)*	1.433 (0.794-2.584)	1.872 (1.138-3.080)*
Western	1.245 (0.683-2.272)	1.244 (0.644-2.404)	1.110 (0.581-2.120)
Mid -western	1.029 (0.501-2.114)	0.709 (0.161-3.122)	1.501(0.905-2.490)
Far-western	1.910 (1.035-3.528)*	2.433 (0.899-6.659)	1.736 (1.059-2.847)*

*:statistically significant. NDHS: Nepal Demographic and Health Surveys.

Model 1: All independent variables of interest shown in Figure 1 (Conceptual framework) were entered in the initial model for pooled data, and 2006 and 2011 survey data. # ANC was replaced by iron (in each models, and the odds ratios were presented in the above mentioned table) as iron supplementation and ANC visits were highly correlated (r = 0.8). Model 2 and Model 3: Effect modification of the survey period were examined including the four interaction terms ANC*Year (F = 2.697; p = 0.069), Iron*Year (F = 0.908; p =0.341 ), Region*Year (F = 0.505; p = 0.732), Education of mothers* Years (F = 2.936, p value = 0.033) separately. As one of the interactions was found statistically significant, we built additional regression models for each year separately; Model 2: Survey 2006 and Model 3: Survey 2011.

Effect modification was assessed by including interaction terms between the indicator variable (representing the survey years) and the significant factors obtained into Model 1 (pooled data). Amongst the four interactions considered, only education of mothers was found to be significantly interacted with years of survey: ANC*Year of survey (F = 2.697; p = 0.069), Iron*Year of survey (F = 0.908; p =0.341 ), Region*Year of survey (F = 0.505; p = 0.732), Education of mothers* Years of survey (F = 2.936, p value = 0.033). As the year of survey significantly modified the effect of one of the determinants on LBW, we built additional regression models stratified by period (year): Model 2 for the 2006 survey and Model 3 for the 2011 survey. Similar to Model 1, ANC visits and iron consumption were statistically significant factors associated with LBW in both 2006 (Model 2) and 2011 (Model 3) surveys. However, maternal education was found significant only for 2006 survey data suggesting the mothers who only gained primary education had two times [95% CI (1.224-3.650)] higher chance of having a LBW infants compared to those who had completed secondary education. In 2011 survey, the effect of region was similar to what we found for the pooled data, i.e. mothers who lived in the Eastern [OR 1.872; 95% CI (1.138-3.080)] and Far-western region [OR 1.736; 95% CI (1.059-2.847)] were more likely to have LBW infants compared to mothers from Central region. Overall, the protective effect of attending ANC visits and iron consumption during pregnancy in preventing LBW was proved in all three models.

## Discussion

The Government of Nepal has committed to achieve Millennium Development Goals (MDG) and has achieved a significant progress in maternal and child survival goals. However, the on-going challenge remains in reducing the stagnant newborn mortality rate as it still remained unchanged since 2006 [23]. LBW is one of the major factors associated with higher newborn mortality in developing countries including Nepal. This is the first study from Nepal which reports the factors associated with LBW based on the data which cover the entire country. This study is based on the national level data that used internationally validated questionnaires with a strong methodology [24]. The comparison in this study gives an indication for future intervention and a benchmark for future comparisons.

This study revealed that the prevalence of LBW has not been significantly reduced over NDHS 2006 and the 2011. Likewise, there was also no increase in the birth weight. The Nepalese mothers, generally, are the cohorts of the children when there used to be a very high under nutrition. Until today, four in ten children aged under five years suffer from underweight or stunting [8,13]. The mother’s status in her father’s house (as a child), and in her husband’s house (as a wife and daughter-in-law) remains lower. This lower status causes lesser use of health services during pregnancy and childbirth, and less priority to maternal nutrition intake. Such chronic under nutrition and lower status as a female in family may have an intergenerational effect on the birth weight of the newborns of Nepal. There has been a greater focus on the issue of child and maternal health than any other health issues in Nepal. However, in contrast, National Safe Motherhood and Newborn Health Long Term Plan (2006-2017) [25] which is a guiding framework for increasing maternal and child survival in Nepal does not provide or recommend on intervention to reduce the current higher LBW prevalence in Nepal. Such lower focus on LBW issue, in part, can explain the lack of improvement in mean birth weight and prevalence of LBW despite having such a high focus on child health.

The association of the use of recommended ANC service and iron supplementation consumption during pregnancy with LBW are two important findings of this study. Both factors were found to be protective against LBW consistently in the pooled, 2006 and 2011 survey datasets. ANC visits are likely to influence in improvements in dietary practices, monitor and encourage recommended weight gain during pregnancy and improve neonatal outcomes [26,27]. The current findings are supported by findings of Huetson et al. [10] that ANC visits were found to be significant protective factors against LBW. In Nepal, mothers are provided with iron-folic acid (combined) supplementations and deworming medication during ANC, and also provided with the advice on rest, and self-care during illness [11]. All these factors are crucial in improving the mother’s health status as well as adequate weight gain during pregnancy [10,28]. The protective effect of iron supplementation is found in this study is consistent with the finding of a previous double blinded intervention study in Nepal [12] which showed an increase in birth weight with such supplementation. A more recent systematic review also reported a reduced prevalence of LBW among the mothers who consumed iron [risk ratio 0.79; 95% CI: 0.61-1.03)], although the difference was not statistically significant [29]. The findings from this study are also in line with a further analysis of Indian national survey, where the authors reported that the consumption of iron supplementation was associated with a lower likelihood of LBW [OR 0.77; 95% CI (0.68-0.87)] [30]. Similarly, consumption of iron supplementation has been reported to be protective against LBW in many studies [31-34]. The physiological mechanism of iron supplementation on birth weight is not clearly understood, however, there are two hypotheses about improvements in birth weight due to iron supplements [35]. First, iron deficiency anaemia leads to changes in norepinephrine, cortisol and corticotrophin resulting in oxidative stress to foetal growth which is reduced by iron supplementation. Second, iron supplementation helps to improve appetite leading to improvement in the overall nutritional status of mother. Improved maternal nutritional status contributes to an increase in infant birth weight. It should be noted that the NDHS collected information on iron supplements; if the mothers had obtained such supplementation from the public health facilities or female community health volunteers, the dosage forms are available only as iron-folic acid form [11]. From the available information it is not possible to explain whether the mothers consumed iron only supplementation or consumed iron-folic acid supplementation, however, the previously published researches mention that the improvement in birth weight was likely to be due to iron [12,30]. Nevertheless, the current study highlights the importance of current iron supplementation in Nepal to reduce the burden of LBW.

Education of mother has been found to be a significant determinant of LBW in the pooled, and 2006 survey data similar to studies from India [7] and Pakistan [36]. The association of the education status of mothers with birth weight can be interpreted in a number of ways. Education is closely related to delayed marriage and child birth thus avoiding adolescent pregnancy. Educated mothers are more likely to be aware of the importance of use of pregnancy care and nutrition care and are more likely to understand health message and more likely to be concerned about their health and nutritional status.

This study reported a higher risk of LBW in the Far-western region and the Eastern regions of Nepal than other regions for pooled and the 2011 survey data. The Far-western region is characterised by its difficult terrain, less access to transportation infrastructure and lesser livelihood opportunity. It is economically and socially under developed area having less access to health care, and food insecurity in high hills and mountain in most times of the year [37]. Such adverse conditions may affect women’s overall health status especially during pregnancy and childbirth leading to poor birth outcomes such as low birth weight, preterm birth, and higher maternal and neonatal mortalities. The reason for a higher burden of LBW in the Eastern region is not clear. Further study is needed to explore the reason. However, such regional differences in child health are not uncommon in South Asia, and have been reported in many studies in Nepal [19,37], India [7] and Pakistan [36].

This study has some limitations. The cross sectional nature of the study prevents it from developing causal inferences. There is possibility that some responses may suffer from recall bias and social desirable responses. While some birth weights were noted on records, many birth weights relied on the mother’s recall of her baby’s weight. Not all the mothers were able to report the birth weight of their child which may have led to an underestimate of the LBW problem. However, comparatively large samples of the children allow this study to reflect national scenario of Nepal.

### Public health implication of the study

The current study indicates the need for targeted interventions aimed at decreasing the high rate of LBW in Nepal. Even if the ANC may not have direct causal link to LBW, it will lead to better nutritional status of mothers and the adoption of healthy behaviours that can influence LBW rates [10,27,38]. Therefore, existing ANC services need to be emphasized. At the community level, mothers need to be supported and encouraged to attend ANC through education and counselling and that this becomes accepted as the social norm and is perceived as benefitting the whole community by reducing LBW and its longer term negative consequences. Given that approximately two in three women aged 15-49 years were anaemic in Nepal [39] and even higher proportion of mothers are likely to suffer from anaemia during pregnancy, the current finding of association of iron consumption with a lower likelihood of having LBW infants suggests that promotion of universal coverage of iron to all the pregnant mothers may bring a significant reduction in LBW in population level. Strengthening existing outreach clinics [11] to increase the access of all pregnant mothers, ensuring that the health facilities are never out of stock of the iron tablet supply and distributing iron tablets through Nepal’s network of the female community health volunteers [40] are feasible options in Nepal. It is advisable that the issue of LBW be integrated in the maternity care guidelines for health professionals in Nepal; specially midwives and nurses who deal closely with women during pregnancy, delivery and the postpartum period [41]. It may help to achieve a reduction in LBW as well as enhance the provision of essential care for the LBW newborn.

## Conclusion

This study found that the LBW prevalence was similar in 2006 and 2011 surveys with no significant change in birth weight. There is an urgent need for intervention to reduce the prevalence of LBW if Nepal is to reduce newborn mortality and keep the current progress on child survival. A greater promotion of utilisation of antenatal care and consumption of iron supplementation is likely to contribute in reduction of LBW in Nepal. Future observational studies should examine other modifiable risk factors of LBW such as medical service utilization, food security and other health related factors.

## Competing interests

The author declares that he has no competing interest.

## Authors’ contributions

VK conceived the study, performed statistical analysis, interpreted the result, and wrote the manuscript. KS and YZ supervised analysis, and contributed in manuscript revision. All authors agreed on the final version of the manuscript.

## Authors' information

VK holds an MPH degree. He has been working in child health programs in Nepal for more than five years. Newborn care and child nutrition is the focus of his work in Nepal and MPH studies. YZ is a senior lecturer in the School of Public Health and teaches in the postgraduate programs. She has an MSc and PhD in statistics. KS is a senior lecturer in the School of Public Health and coordinates the MPH/DrPH programs. She has an MSc and PhD in Behavioural Sciences.
